# Tibial Osteomyelitis Caused by *Morganella morganii* After External Fixation for Limb Length Discrepancy in a Pediatric Patient: A Case Report and Literature Review

**DOI:** 10.5435/JAAOSGlobal-D-22-00171

**Published:** 2022-12-19

**Authors:** Neal Mody-Bailey, Ekene Uchenna Ezeokoli, Jaclyn Hill

**Affiliations:** From the Baylor College of Medicine, Houston, TX (Mody-Bailey); the Oakland University William Beaumont School of Medicine, Rochester, MI (Ezeokoli) and the Department of Orthopaedics and Scoliosis Surgery, Texas Children's Hospital, Houston, TX (Ezeokoli, Dr. Hill).

## Abstract

*Morganella morganii* is a facultative, anaerobic rod Gram-negative enteric bacterium. Few cases are documented of musculoskeletal infection. We present a case of a 9-year-old boy with osteomyelitis 1 year after index external fixation for leg length discrepancy. Our patient initially presented with wound drainage at his distal medial tibia fixation site but had negative radiographs. Initial antibiotic treatment failed after 1-month follow-up, and cultures revealed *M. morganii*. He underwent incision and drainage with external fixator removal, and the antibiotic regimen changed after a susceptibility panel. Symptoms were alleviated after 30 days with the new antibiotic regimen, and the patient was put back on his original schedule for limb lengthening through external fixation. A new methicillin-sensitive *Staphylococcus aureus* occurred at the same site 2.5 years later but was easily treated, and the 3-year follow-up showed no other recurrences or complications.

Morganella morganii is a facultative, anaerobic rod Gram-negative enteric bacterium, first isolated in 1906 by Morgan et al*.*^1^ It is a rare opportunistic pathogen most associated with wound, soft tissue, and urinary tract infections, but there have been reports of sepsis, ecthyma, endophthalmitis, chorioamnionitis, and pericarditis.^2^ Drug resistance of *M. morganii* has increased over recent years increasing pathogenicity^3^ and clinical treatment failure.^4,5^ Mortality rates are relatively high in some reports.^2^

Osteomyelitis is caused by a wide variety of pathogens, most commonly various strains of *Staphylococcus aureus*, *Streptococci*, *Enterobacteriaceae*, *and Pseudomona.*^6^ As of the time of literature review, there have only been a handful of documented cases of osteomyelitis secondary to *M. morganii*. We describe the clinical course of an infection in a pediatric patient after external fixation surgery for leg length discrepancy at a tertiary health facility.

## Case

A 9-year-old boy, with a history of limb length discrepancy, presented with medial drainage of the right distal tibia at his surgery incision site for 10 days. The patient had an extensive surgical history including compartment syndrome and right lower leg necrotizing fasciitis at 5 months of age after an insect bite requiring multiple debridements. This resulted in a right tibial physeal arrest causing notable leg length discrepancy and subsequent tibia and fibula lengthening/shortening surgery through external fixation at age 8 years.

The patient denied fever, changes in weight, or limitation in activity. No evidence of foul smells, cellulitis, or abscesses was found. Physical examination showed a mild sinus along the medial aspect of the medial malleolus expressing clear fluid. Imaging showed no evidence of osteomyelitis. Cultures were taken, and the patient was started on a 21-day trial of per oral (PO) clindamycin 75 mg/5 mL 3× daily. At the 1-month follow-up, the patient had completed the full regimen of antibiotics; however, serosanguinous drainage was still noted within the incision sight at the medial malleolus. The patient continued to deny fever, limitation to activity, or systemic illness. Imaging again showed no evidence of osteomyelitis, loosening of implants, or changes in position. The patient was scheduled for débridement and irrigation of the area. Wound and bone cultures grew *M. morganii*. Laboratory results obtained 2 weeks after his follow-up showed normal erythrocyte sedimentation rate and C-reactive protein, consistent with chronic infection, and a normal blood urea nitrogen/Cr, aspartate transaminase/alanine transaminase (AST/ALT), and complete blood count with differential with no evidence of leukocytosis.

**Figure 1:**
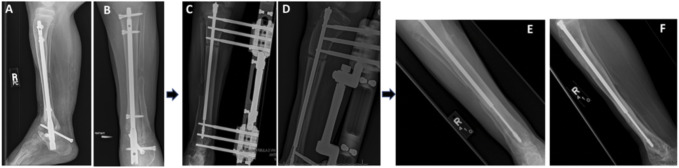
Patient right tibia and ankle radiographs demonstrating timeline of infection. **A**, Lateral and (**B**) AP radiographs at the time of *M. morganii* infection demonstrating persistent irregularity to the distal right tibia and an osteotomy gap but with no lucent lesions or signs of osteomyelitis. **C**, AP and (**D**) oblique radiographs 1.4 years after *M. morganii* infection. **E**, AP and (**F**) lateral radiographs 2.5 years after *M. morganii* during our patient's methicillin-sensitive *S. aureus* (MSSA) infection demonstrating lucency of distal tibia.

It was decided that the patient should have the intramedullary nail from his original limb lengthening surgery removed and be treated for chronic osteomyelitis, regardless of radiological evidence (Figure 1). The patient was sent home on PO clindamycin 150 mg 3x daily (TID) but was subsequently switched to PO cefdinir 250 mg/5 mL 2x daily (BID) × 30 days for the treatment of chronic osteomyelitis, per infectious disease recommendation (Table 1). Cefdinir was used due to a history of penicillin allergy. One month later, the patient underwent removal of the deep rod implant with no complications. At 1 and 6 months postoperatively, the patient showed drastic improvement with no incidence of drainage, fever, or limitation of activity. Imaging at 6 months postoperatively showed no changes with his leg length discrepancy at 5.5 cm.

**Table 1:** *Morganella morganii* Antibiotic Susceptibility in Our Patient

Antibiotic	MIC (µg/mL)	Interpretation
Cefotaxime	0.032	Susceptible
Ciprofloxacin	2.0	Intermediate
Levofloxacin	1	Susceptible
Piperacillin/tazobactam	1.0	Susceptible
Cefoxitin	32	Resistant
Tobramycin	2	Susceptible
Amikacin	≤2	Susceptible
Ampicillin	≥32	Resistant
Gentamicin	≥16	Resistant
Meropenem	≤0.25	Susceptible
Piperacillin	≤4	Susceptible
Trimeth sulfa	≥320	Resistant

**Figure 2:**
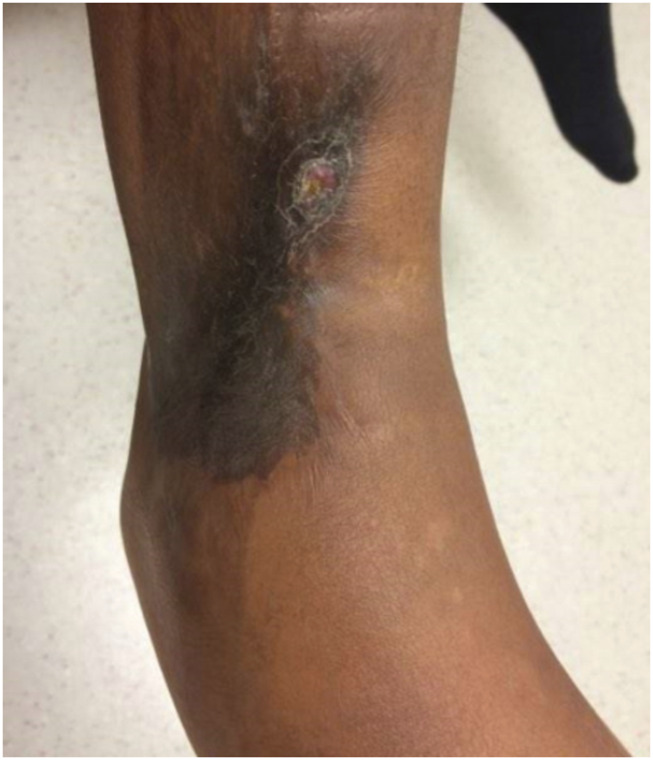
Photograph showing the site of mediolateral drainage on distal right leg during methicillin-sensitive *S. aureus* (MSSA) infection 2.5 years after index *M. morganii* infection.

The patient was then continued on his original schedule for fixation for leg length discrepancy, requiring one additional tibia and fibula lengthening/shortening with an external fixation procedure. Two and a half years after the initial *M. morganii* infection, the patient acquired a second infection secondary to methicillin-sensitive *S. aureus* at his lateral ankle with radiological evidence showing lucency of the bone (Figure 2). The patient required irrigation and débridement of the area, removal of hardware, and PO clindamycin 300 mg TID × 30 days. The patient was followed for an additional 3 years. No other recurrences or complications were found related to the original infection.

## Discussion

*Morganella morganii* is a rare opportunistic pathogen, typically causing urinary tract and postoperative wound infections.^7^ This case presents a highly unusual etiology for osteomyelitis. There have only been a handful of cases reported on *M. morganii* and even fewer causing osteomyelitis (Table 2). The literature review showed that most cases of *Morganella* osteomyelitis occurred in the distal lower extremities near a past surgical or traumatic site. Our case was unique because symptomatic evidence of infection was not evident until 1 year after surgery. *M. morganii* is widespread throughout the environment and naturally inhabits our intestinal tract.^7^ Due to the time gap from operation to symptomatic presentation, community acquisition with hematogenous inoculation is a plausible theory for etiology.

**Table 2:** Literature on *Morganella morganii* Osteomyelitis

Publication Year	Location	Age/Sex	Infection Location	Treatment Regimen	Outcome
1985^8^	United States	61/f	Femur	I&D, aztreonam × 50 d	Above knee amputation day 22, 16-week follow-up
1998^9^	United States	57/m	Foot, cuneiform	I&D, IV gentamicin	Resolution with incomplete wound healing on 1-year follow-up
2004^10^	Spain	79/m	Rib	Ciprofloxacin × 6 wk	Resolution in 3 wk, follow-up n/a
2009^11^	France	n/a	Foot	n/a	n/a
2009^12^	China	44/m	Distal femur	I&D, IV imipenem × 5 wk	Resolution with 3 months of follow-up
2016^13^	United States	50/m	2nd toe, 2nd metatarsal head	Amputation, IV ertapenem × 6 wk	Follow-up n/a
2016^14^	India	56/m	Proximal tibia	I&D, IV piperacillin-tazobactam + amikacin × 6 wk	Resolution, follow-up n/a
2019^15^	United States	16 mo/f	Talus	I&D, oral cefixime × 3 wk	Resolution with 1-year follow-up
2019^16^	Germany	n/a	Foot	n/a	n/a
2019^17^	Turkey	56/m	Distal femur, proximal tibia	I&D, IV piperacillin-tazobactam	Inpatient cardiopulmonary arrest
2019^18^	China	58/f	4th digit, proximal phalanx	I&D, IV piperacillin	Finger amputation day 7, 3-month follow-up
2022 (this case)	United States	9/m	Distal tibia	I&D, PO cefdinir × 30 d	Resolution, follow-up 6 months

n/a = not available

A article by De et al.^14^ showed that strains of *M. morganii* have the propensity for biofilm production and removal of biofilm is essential for adequate treatment. In the absence of biofilm and with proper antibiotic penetrance, most cases showed complete resolution of infection without the need for amputation. The antibiotic regimen varied across cases, most treated with broad spectrums, covering gram positives, negatives, and anaerobes. In this case, once *Morganella* was isolated, antibiotic treatment was transitioned from clindamycin to a third-generation cephalosporin (cefdinir). The literature review showed one other case choosing to make the same transition in the setting of a foot abscess colonized by *Morganella*; in this case, the patient had complete resolution within 1 year of management.^12^ Interestingly, in our case, there was a lack of radiological evidence for osteomyelitis and the decision for treatment was based on symptomatology and microbiological evidence. One other case report documented the lack of initial plan radiograph evidence; however, follow-up MRI showed radiological features of osteomyelitis.^15^ Plain radiographs are the best screening measure for chronic osteomyelitis; however, up to 50% to 75% of the bone matrix must be destroyed for the radiograph to show lytic lesions.^19^ MRI should be considered if radiological evidence is wanted because the pathogenesis of osteomyelitis caused by atypical pathogens may not mirror common etiologies. In addition, external fixation has a known high risk of pin tract infection, though superficial. It is important to be wary when using intramedullary nailing in a limb lengthening capacity due to risk of spread to the whole bone.

In this report, we described a unique case of chronic osteomyelitis caused by the rare pathogen, *M. morganii.* The results of our case report and literature review can be summarized by three general findings. (1) *Morganella* may result in subtle changes in the bone matrix making it difficult to appreciate on radiological imaging. In cases where radiological evidence for osteomyelitis is wanted, advanced imaging such as MRI should be considered. (2) Strains of *Morganella* have the capability of producing biofilm. In the setting of implanted hardware, *Morganella* can be refractory to routine debridements and antibiotics. Removal of hardware should be considered for eradication of the pathogen. (3) Treatment for *Morganella* typically revolves around broad spectrum antibiotics; of the limited incidents, cefixime and piperacillin-tazobactam have shown to result in complete resolution.
